# Functional Alteration of Natural Killer Cells and Cytotoxic T Lymphocytes upon Asbestos Exposure and in Malignant Mesothelioma Patients

**DOI:** 10.1155/2015/238431

**Published:** 2015-06-16

**Authors:** Yasumitsu Nishimura, Naoko Kumagai-Takei, Hidenori Matsuzaki, Suni Lee, Megumi Maeda, Takumi Kishimoto, Kazuya Fukuoka, Takashi Nakano, Takemi Otsuki

**Affiliations:** ^1^Department of Hygiene, Kawasaki Medical School, 577 Matsushima, Kurashiki 701-0192, Japan; ^2^Laboratory of Functional Glycobiochemistry, Department of Biofunctional Chemistry, Division of Agricultural and Life Science, Graduate School of Environmental and Life Science, Okayama University, Okayama 700-8530, Japan; ^3^Okayama Rosai Hospital, Okayama 702-8055, Japan; ^4^Department of Respiratory Medicine, Hyogo College of Medicine, Nishimomiya 663-8501, Japan; ^5^Department of Medical Oncology, Faculty of Medicine, Kinki University, Sayama 589-8511, Japan

## Abstract

Malignant mesothelioma is caused by exposure to asbestos, which is known to have carcinogenic effects. However, the development of mesothelioma takes a long period and results from a low or intermediate dose of exposure. These findings have motivated us to investigate the immunological effects of asbestos exposure and analyze immune functions of patients with mesothelioma and pleural plaque, a sign of exposure to asbestos. Here, we review our knowledge concerning natural killer (NK) cells and cytotoxic T lymphocytes (CTL). NK cells showed impaired cytotoxicity with altered expression of activating receptors upon exposure to asbestos, while induction of granzyme^+^ cells in CD8^+^ lymphocytes was suppressed by asbestos exposure. It is interesting that a decrease in NKp46, a representative activating receptor, is common between NK cells in PBMC culture with asbestos and those of mesothelioma patients. Moreover, it was observed that CD8^+^ lymphocytes may be stimulated by some kind of “nonself” cells in plaque-positive individuals and in mesothelioma patients, whereas CTL in mesothelioma is impaired by poststimulation maintenance of cytotoxicity. These findings suggest that analysis of immunological parameters might contribute to the evaluation of health conditions of asbestos-exposed individuals and to a greater understanding of the pathology of malignant mesothelioma.

## 1. Introduction

Inhalation of naturally occurring particles and fibers causes not only pulmonary fibrosis following an inflammatory response, but also tumor and autoimmune diseases. To date, we have focused on and examined the effect of asbestos exposure on the functions of various kinds of immune competent cells. These studies confirmed that functional decreases in T helper (Th) cells, natural killer (NK) cells, and cytotoxic T lymphocytes (CTLs) were caused by exposure to asbestos, decreases that were also partly observed in patients with malignant mesothelioma, following examination of cell lines and primary cells cultured with asbestos and analyzing cells prepared from the peripheral blood of patients (Table 1) [1–8]. Recently, we have concentrated on the analysis of CTL function in individuals exposed to asbestos and in patients with malignant mesothelioma and found interesting similarities and differences between the groups [9]. These studies give us the opportunity to think in an integrated manner regarding alteration of tumor immunity, and the role played by NK cells and CTLs upon asbestos exposure and in mesothelioma patients. Therefore, here we first show findings concerning NK cells and then investigate CTLs as found in a cell culture exposed to asbestos as well as in individuals exposed to asbestos and patients with malignant mesothelioma. Before discussing these subjects, we first describe the background of our studies, various aspects of asbestos, malignant mesothelioma, and the relationship between asbestos exposure and immune function.

## 2. Asbestos, Malignant Mesothelioma, and Immune Function

**Figure 1:**
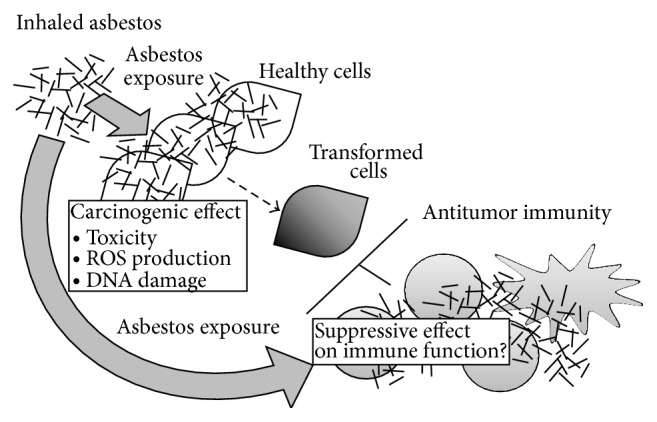
Possible effect of asbestos exposure on antitumor immunity. It is illustrated that there might be a suppressive effect of asbestos exposure on antitumor immunity in the pathology of tumor diseases caused by exposure to asbestos. It is well known that asbestos has a carcinogenic effect, but the development of malignant mesothelioma takes a long time after exposure to asbestos, suggesting the existence of effective antitumor immunity and subsequent impairment caused by asbestos exposure.

Asbestos is a kind of naturally occurring mineral fiber that has valuable physical and chemical characteristics including flexibility, as well as fire and heat resistance, which has resulted in the enormous use of asbestos globally. However, in the latter part of the 20th century many reports established that inhalation of asbestos causes malignant mesothelioma, which marked asbestos as one of the representative carcinogenic materials [10–16]. Malignant mesothelioma begins in mesothelial cells covering the inner surface of pleural, pericardial, and peritoneal cavities, as well as the tunica vaginalis, and pleural mesothelioma is the major condition [17]. Asbestos is classified as a Group 1 carcinogen by the International Agency for Research on Cancer (IARC), and it is thought that almost all cases of malignant mesothelioma are caused by exposure to asbestos. Asbestos causes cellular toxicity and mutagenicity and induces the generation of reactive oxygen species (ROS). In addition, it is known that the amounts of oxidized pyrimidine and alkalized nucleic acid base components correlate with the period of exposure to asbestos and that intratracheal instillation of asbestos induces an increase in the mutation frequency of lung DNA in rats [17–21]. However, the relationship between asbestos and malignant mesothelioma cannot be attributed to a “dose-dependent relationship,” which is regarded as a general rule in toxicology. It is thought that malignant mesothelioma is caused by a relatively low or intermediate dose of asbestos exposure, whereas a high dose of exposure causes asbestosis [22]. It is also known that malignant mesothelioma occurs even in people exposed environmentally to asbestos. In addition, it takes about 40 years to develop malignant mesothelioma after the initial exposure to asbestos. These findings suggest that mesothelioma may not be caused just by the direct effect of asbestos on mesothelial cells, in which asbestos exposure might exert some kind of alternative effect on the body and allowed us to consider that one possible candidate is its effect on immune function (Figure 1). Inhaled asbestos reaches the pleural cavity through the trachea, bronchus, and pulmonary alveoli, and some asbestos arrive at the regional lymph nodes. Accumulation of asbestos in lymph nodes was observed in people exposed to asbestos nonoccupationally and occupationally [23, 24]. Dodson et al. reported that the total amount of asbestos in the lung was quite low, whereas most cases having asbestos in the lymph nodes showed larger amounts of asbestos in the nodes than in the lung [23, 24]. Thus, immune competent cells may have contact with asbestos not only in nonlymphoid tissue and the area of pulmonary parenchyma and pleural cavity with the inflammatory response, but also in lymphoid tissue of bronchoalveolar, mediastinal, and intercostal nodes, and so on.

## 3. Activating Receptors on NK Cells

**Figure 2:**
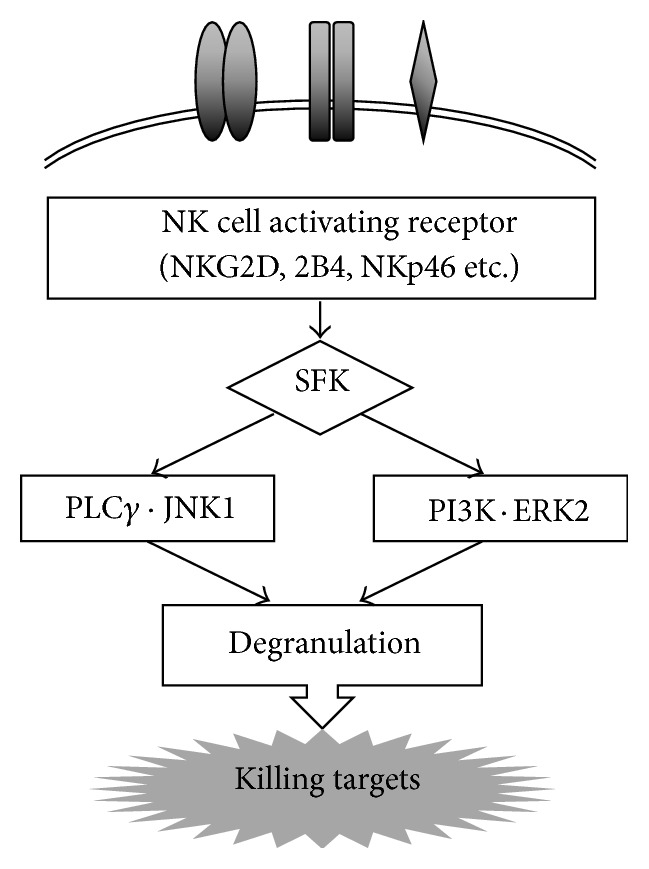
NK cell activating receptors and signal transduction leading to killing targets. NK cells recognize target cells by various kinds of activating and inhibitory receptors. Activation uses common machinery that induces cytotoxicity for targets. The bindings of activating receptors with each ligand transduce through the Src family kinase- (SFK-) dependent phosphoinositide-3 kinase (PI3K) → extracellular signal-regulated kinase 2 (ERK2) pathway and the phospholipase C*γ*→ c-Jun N-terminal kinase 1 (JNK1) pathway. Finally, degranulation is induced, by which perforin and granzymes in cytotoxic granules are released and work on target cells to induce apoptosis.

Our study focused on and examined the expression of activating receptors on the surface of NK cells, which play a crucial role in recognition for target cells leading to induction of cytotoxicity. Instead of antigen-specific receptors such as the T cell receptor and immunoglobulin for T and B cells, NK cells express various kinds of activating and suppressive receptors [25, 26]. Suppressive and activating receptors on NK cells recognize MHC class I and the ligands derived from infected viruses and tumor cells to contribute to suppression and activation of cytotoxicity, respectively. Some of the killer cell immunoglobulin-like receptors (KIRs) and heterodimers CD94 and NKG2A or NKG2B are suppressive receptors. On the other hand, the homodimer of NKG2D, 2B4, which is a member of the signaling lymphocyte activation molecule (SLAM) family, and NKp46, a member of the natural cytotoxicity receptor (NCR) family, are receptors that play a role in the induction of cytotoxicity. It has been found that activating receptors employ the same pathway of intracellular signal transduction [27]. After binding of ligands, degranulation of cytotoxic granules including perforin and granzymes is induced through the Src family kinase- (SFK-) dependent phosphoinositide-3 kinase (PI3K) → extracellular signal-regulated kinase 2 (ERK2) pathway and the phospholipase C*γ*→ c-Jun N-terminal kinase 1 (JNK1) pathway. Perforin and granzymes released by degranulation cause target cells to undergo apoptosis (Figure 2).

## 4. Impaired Cytotoxicity and Altered Expression of Activating Receptors in an NK Cell Line Exposed to Asbestos

We began examining the effect of asbestos exposure on cell lines. The human NK cell line of YT-A1 was cultured with continuous exposure to chrysotile B (CB) asbestos at 5 *μ*g/mL, named the YT-CB5 subline and was then examined periodically concerning cytotoxicity for K567 cells and expression of cell surface receptors, with results being compared to those of the control subline of YT-Org. Initially, the effect of asbestos exposure on viability of the cell line was checked and 5 *μ*g/mL of CB was chosen and utilized as a concentration having no effect on cell growth and apoptosis. Although YT-CB5 showed a normal level of cytotoxicity comparable to YT-Org until 1 month after the start of culture, it showed impaired cytotoxicity after around 5 months [5]. In accord with the impaired cytotoxicity, YT-CB5 showed decreases in cell surface expression of NKG2D and 2B4, whereas NKG2A and CD94 showed no changes in expression. Although it is known that cytotoxicity against K562 cells is independent of 2B4, the decrease in 2B4-dependent cytotoxicity in YT-CB5 was confirmed by a reverse antibody-dependent cell-mediated cytotoxicity (ADCC) assay. Moreover, YT-CB5 showed the decrease in phosphorylation of ERK1/2 following incubation with K562 cells, and the SFK inhibitor of pp2 or the PI3K inhibitor of wortmannin caused the decrease in phosphor-ERK1/2 of YT-Org. In addition, YT-CB5 also showed a low level of phosphor-ERK1/2 under stimulation with antibody to NKG2D [6]. Thus, it was found that asbestos exposure causes impairment of cytotoxicity with altered expression of activating receptors in NK cells.

## 5. Decrease in NKp46 on NK Cells in Culture upon Asbestos Exposure and in Patients with Malignant Mesothelioma

**Figure 3:**
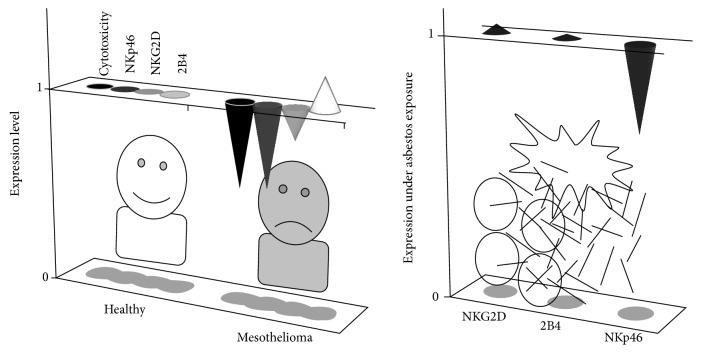
The characteristic decrease of NKp46 on NK cells shared by* in vitro* asbestos exposure and malignant mesothelioma patients. Peripheral blood NK cells in 7 patients with malignant mesothelioma showed decreased cell surface NKp46, but not NKG2D or 2B4, among activating receptors, compared with 10 healthy individuals, and it is interesting that this was also shown by NK cells in PBMCs cultured with asbestos. The relative alterations of expression level are shown.

After the study of the cell line exposed to asbestos, we examined the function of peripheral blood NK cells in patients with malignant mesothelioma. Peripheral blood mononuclear cells (PBMCs) prepared from peripheral blood were assayed for cytotoxicity against K562 cells and the expression level of activating receptors on the cell surface of NK cells, and results were compared between healthy and mesothelioma individuals. To evaluate the lytic activity of a given cell number, the cytotoxicity per 5000 NK cells was calculated from the percentage of NK cells in PBMCs. Mesothelioma patients showed lower cytotoxicity of NK cells than healthy individuals, and also exhibited alteration in expression of activating receptors in their NK cells, which differed from YT-CB5. The NK cells of mesothelioma patients exhibited a characteristic decrease in expression of NKp46, whereas NKG2D and 2B4 showed normal expression (Figure 3) [5]. PBMCs were then cultured in media supplemented with IL-2 and exposed to CB at 5 *μ*g/mL for 7 days and assayed for the expression of activating receptors on the cell surface of NK cells. As shown by NK cells of mesothelioma patients, NK cells showed a decrease in NKp46 in the culture upon CB exposure, whereas NKG2D and 2B4 did not differ from the control culture (Figure 3). In addition, glass wool, which represents a man-made mineral fiber and a substitute for asbestos, did not cause such an alteration in expression of activating receptors, unlike CB asbestos. It was therefore interesting to discover that peripheral blood NK cells in mesothelioma patients showed a characteristic decrease in cell surface NKp46 with low cytotoxicity, similar to that of NK cells in the culture with asbestos, suggesting the possibility that impairment of NK cell function might be caused by inhaled asbestos and may be related to the pathology of malignant mesothelioma.

## 6. Relationship between Cytotoxicity, Expression of Activating Receptors, and Signal Transduction in NK Cells

**Figure 4:**
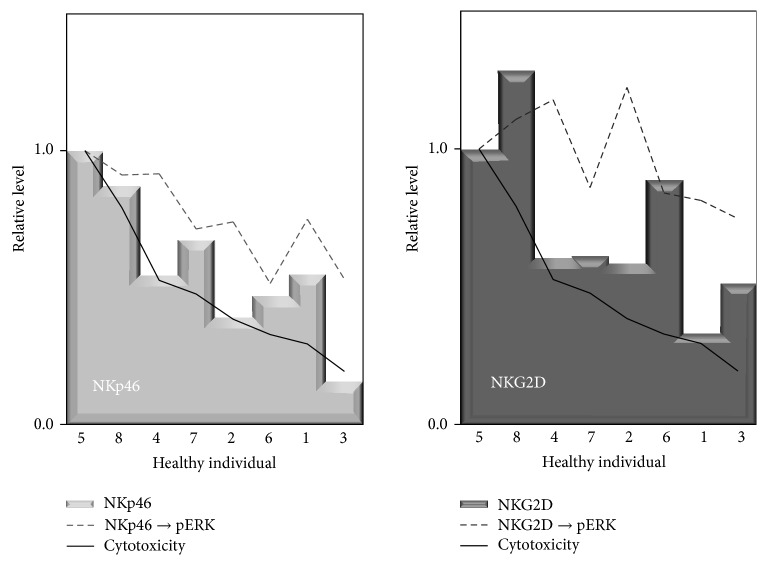
Relationship among cytotoxicity, expression of NKp46 or NK2D, and ERK phosphorylation. When the NK cells of healthy individuals were analyzed, an individual with high cytotoxicity showed high expression of NKp46 and high phosphorylation of ERK induced through NKp46, and there was also a similar relationship among cytotoxicity, NKG2D expression, and ERK phosphorylation. These findings suggest that low expression of NKp46 may be attributed to decreased cytotoxicity of NK cells in patients with malignant mesothelioma. The levels in each individual relative to an individual with the highest cytotoxicity are shown.

To examine the relationship between low levels of cytotoxicity and activating receptors, peripheral blood NK cells were isolated from the PBMCs of healthy individuals and assayed regarding cytotoxicity for K562 cells, cell surface expression of activating receptors, and phosphorylation of ERK1/2 after stimulation with antibodies to receptors, and results were compared between individuals. When individuals were put in descending order of cytotoxicity, an individual with high cytotoxicity showed high expression of NKp46 and NKG2D, whereas another with low cytotoxicity showed the opposite trend (Figure 4). In accord with this finding, an individual with high cytotoxicity showed a high level of phosphor-ERK1/2 following stimulation with antibodies to NKp46 or NKG2D, whereas another with low cytotoxicity showed a low level of phosphorylation of ERK1/2 [6]. In contrast, the expression level of 2B4 and the phosphorylation level of EKR1/2 following stimulation with 2B4 did not show such a relationship with cytotoxicity. These findings indicate that expression levels of NKG2D and NKp46 are related to the degree of cytotoxicity induced by stimulation with those receptors through signal transduction downstream of the receptors, suggesting that decreased cytotoxicity of NK cells in mesothelioma patients might be attributed to low expression of NKp46.

## 7. Cytotoxic T Lymphocytes and Inhalation Exposure to Asbestos

In antitumor immunity, CD8^+^ T lymphocytes play a more crucial role in cytotoxicity against target cells in an antigen-specific manner, together with the natural cytotoxicity of NK cells [28]. CTLs also utilize the same tools to injure targets such as NK cells, in which perforin and granzymes are released from CTLs into an intercellular space and induce apoptosis of target cells [29]. However, CD8^+^ T lymphocytes have to be selected clonally and proliferate and differentiate into functional CTLs in order to exert matured cytotoxicity for targets [30]. The differentiation of functional CTLs is a very complex event, in which various kinds of immune cells contribute to CTL differentiation. At first, dendritic cells transfer antigen to lymphoid organs as regional lymph nodes, where they or node-resident dendritic cells are ready to present antigen for T cells as antigen presenting cells (APC) [31]. Naïve CD8^+^ T lymphocytes then migrate into the nodes and communicate with APC, and those having specificity for the antigen presented are chosen [32]. Moreover, CD4^+^ T lymphocytes capable of recognizing the same antigen also have to migrate into the nodes and communicate with APC to lead to differentiation of functional CTLs [33]. Thus, the lymph node is a place where immune cells necessary for CTL differentiation meet and communicate with each other, while inhaled asbestos also migrates into regional nodes and accumulates there as mentioned above. These findings indicated that inhaled asbestos might affect differentiation of functional CTLs and motivated us to examine this possibility.

## 8. Effect of Asbestos on Induction of Cytotoxic T Lymphocytes by Mixed Lymphocyte Reaction

We thus attempted to investigate the effect of asbestos on acquired immunity in the antitumor response using the mixed lymphocyte reaction (MLR), an experimental method to induce cell-mediated acquired immunity using an allogeneic set of PBMCs or lymphoid cells, and an easy tool to mimic* in vitro* induction of CTL function from naïve CD8^+^ T cells. PBMCs were cultured with a stimulator of allogeneic PBMCs upon exposure to CB asbestos, and examined for the characteristics and allogeneic cytotoxicity of CD8^+^ T cells [7]. CB exposure suppressed the increase in cell number of CD8^+^ lymphocytes, which when sorted from the culture showed a decrease in cytotoxicity against allogeneic targets compared with those from the control culture. CD8^+^ lymphocytes from the CB-exposed culture also showed low percentages of intracellular granzyme B and IFN-*γ* and cell surface CD25 and CD45RO, and a high percentage of CD45RA. In addition, suppressed cell proliferation of CD8^+^ lymphocytes upon exposure to CB was also confirmed by the CFSE labeling method. In contrast, those lymphocytes did not differ in apoptosis from those of the control group. Moreover, the productions of TNF-*α* and IFN-*γ* in the supernatant from the CB-exposed culture were low, whereas IL-2 did not differ in production. These findings therefore indicate that the induction of CTL function in MLR was suppressed by asbestos, and it was found that asbestos exposure has the potential to exert a suppressive effect on CTL induction following antigen stimulation.

## 9. Functional Properties of CD8^+^ Lymphocytes in Patients with Pleural Plaque and Malignant Mesothelioma

**Figure 5:**
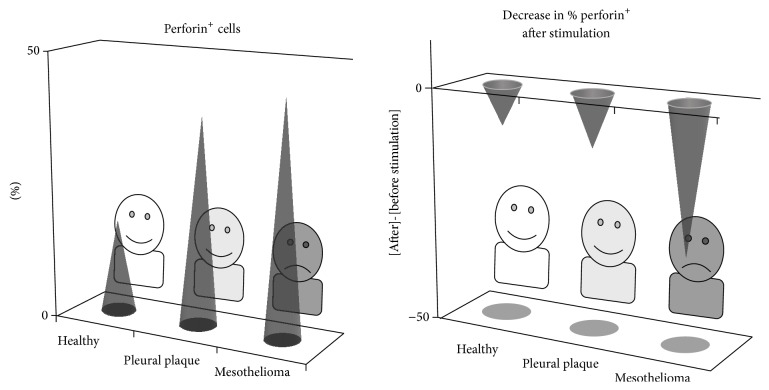
CTL function in patients with malignant mesothelioma and individuals positive for pleural plaque. Both 16 plaque-positive individuals and 14 mesothelioma patients showed a high percentage of perforin^+^ cells in CD8^+^ lymphocytes, compared with 16 healthy volunteers, whereas a decrease after stimulation was observed in mesothelioma. These findings indicate that CD8^+^ lymphocytes are stimulated by some kind of “nonself” cells in both plaque-positive individuals and mesothelioma patients, and that poststimulation maintenance of cytotoxicity is impaired in mesothelioma.

Malignant mesothelioma is attributed to asbestos exposure, which can be determined by examination for pleural plaque using image analyses involving X-ray and CT-scan methods. Pleural plaque is an objective sign of previous asbestos inhalation, and is known to be whitish, sharply circumscribed, fibrous, hyaline, sometimes calcified, forms patches involving parietal pleura, and is regarded as harmless [34]. Our recent analysis of peripheral blood CD8^+^ lymphocytes in individuals positive for pleural plaque and patients with malignant mesothelioma revealed the similarities and differences between these groups. Individuals in the pleural plaque and malignant mesothelioma groups showed higher percentages of perforin^+^ cells and CD45RA^−^ cells in fresh CD8^+^ lymphocytes than healthy individuals. However, patients in the mesothelioma group showed a decrease in perforin^+^ cells following stimulation with PMA and ionomycin, whereas most of the healthy and plaque-positive individuals retained those cells after stimulation (Figure 5) [9]. The decrease in cells positive for intracellular perforin following stimulation might have been attributed to enhanced degranulation of cytotoxic granules, indicating increased cytotoxicity in mesothelioma, since degranulation is a process that releases perforin and granzymes, which act as factors to injure target cells. However, we confirmed that the CD8^+^ lymphocytes did not show an increase of cell surface CD107a, a representative marker of degranulation, following stimulation. Thus, it was clarified that patients with malignant mesothelioma have characteristics of impairment in stimulation-induced cytotoxicity of peripheral blood CD8^+^ lymphocytes. Additionally, it is also important that they showed a similar alteration of function, namely, an increase in perforin^+^ cells, compared to CD8^+^ lymphocytes in plaque-positive individuals, which suggests that such a characteristic might be related with inhalation exposure to asbestos.

## 10. Significance of Our Study Results

As described above, our studies demonstrated that asbestos exposure has the potential to cause suppressed function of NK cells and CTLs. Malignant mesothelioma is caused by exposure to asbestos, but its development is limited by the parts that have been exposed to asbestos, suggesting the existence of effective antitumor immunity against transformed cells at an initial phase in the body of individuals exposed to asbestos. In addition, it is well known that asbestos-exposed individuals take a very long time to develop malignant mesothelioma after exposure, suggesting that antitumor immunity fought transformed cells until the individual began to suffer from malignant mesothelioma. These findings highlight the importance of the monitoring and intervention of immune function in asbestos-exposed people.

In fact, our study identified one appealing candidate for antitumor immunity in relation to asbestos exposure and malignant mesothelioma, namely, NKp46. NK cells in PBMCs showed decreased cell surface expression of NKp46 following exposure to asbestos, which was also shown by patients with malignant mesothelioma. Although our study using the cell line showed alteration in expression of activating receptors in a different manner, in which NKG2D and 2B4 decreased, these findings indicated that the decrease in activating receptors is attributed to low cytotoxicity through a decrease in signal transduction downstream of those receptors, and allowed us to understand that expression of activating receptors should be examined for primary cell cultures and specimens of malignant mesothelioma. It is interesting that the expression of activating receptors on both NK cells of asbestos-exposed PBMCs and patients with malignant mesothelioma is altered in a characteristic manner and is similar between these groups, in which there is a decrease of NKp46 but not NKG2D or 2B4, which suggests a relationship between the decrease in NKp46, asbestos exposure, and malignant mesothelioma. NK cells play a primary role in cytotoxicity against nonself targets in innate immunity before lymphocytes specific for those targets are clonally selected, proliferate, and acquire fully matured cytotoxicity. NKp46 might therefore be a useful tool for the evaluation of health conditions in asbestos-exposed individuals.

In addition, asbestos exposure suppressed development of CTL function during MLR, in which CD8^+^ lymphocytes showed decreases in cytotoxicity and the percentage of intracellular granzyme B. These observations allowed us to speculate that CD8^+^ lymphocytes in patients with malignant mesothelioma might show a decrease in granzymes or perforin similar to that shown by the MLR culture. However, there were no decreases in perforin or granzyme, but rather an increase in perforin in fresh CD8^+^ lymphocytes from individuals with malignant mesothelioma, as well as those with pleural plaque, when compared with healthy individuals. These findings appear to be paradoxical, but careful discussion leads to clarification. As mentioned above, CTL function differs in induction and maturation from NK cell function. The former is induced by antigen stimulation, whereas the latter is ready to injure targets without stimulation. It is important to note in the results obtained from the analysis of blood specimens that CD8^+^ lymphocytes showed an increase of perforin in plaque-positive individuals, which had been exposed to asbestos but did not develop any tumors. This means that they have some kind of “non-self” cells prior to tumors, probably caused by exposure to asbestos, which stimulate immune responses in the body. That explanation helped us to realize that both fresh CD8^+^ lymphocytes of pleural plaque and malignant mesothelioma show such a similar character. On the other hand, healthy individuals have no stimulation with “non-self” cells, including no exposure to asbestos. Therefore, it is difficult to compare healthy and plaque-positive individuals as control and asbestos-exposed cultures in MLR, respectively, which could not have been anticipated before the study was performed. However, it is noteworthy that CD8^+^ lymphocytes in plaque-positive individuals show an increase in perforin, suggesting that they are specifically fighting against “nonself.”

**Figure 6:**
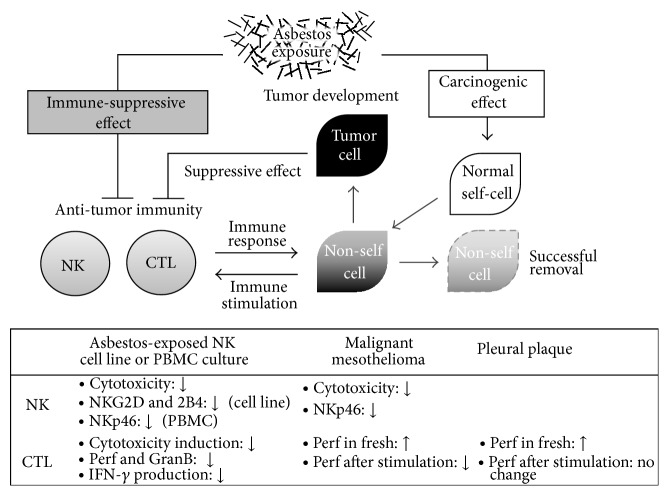
Summary of our study results and discussions. It is illustrated that asbestos exposure may exert not only a carcinogenic effect, but also an immune-suppressive effect, and that there might be an interaction between antitumor immunity and “nonself” cells that results either in the successful removal of these cells or the development of tumor. Our study results are summarized below. Abbreviations: Perf, perforin; GranB, granzyme B.

Furthermore, it is interesting that the CD8^+^ lymphocytes in patients with malignant mesothelioma showed characteristic impairment, in which the percentage of perforin^+^ cells decreased after stimulation, even though it was as high as that of plaque-positive individuals before stimulation. These findings suggest that such impairment in CTL function might be related to the pathology or development of malignant mesothelioma. We can consider the two scenarios for impaired CTL function in malignant mesothelioma. The first may be caused just by the immune-suppressive effect of tumor cells after the onset of malignant mesothelioma. The second may be caused by the immune-suppressive effect of asbestos exposure before malignant mesothelioma, as suggested by results obtained from our experiment using the MLR culture. Although the study concerning CD8^+^ cells did not find any impairment of function in individuals with pleural plaque, we have reported that CD4^+^ T lymphocytes in individuals with pleural plaque showed a decrease in cell surface expression of CXCR3, a chemokine receptor dominantly expressed on Th1 cells [3], which supports the second aforementioned scenario. However, further studies are needed to conclude this matter. In either case, it is clear that CTL function is impaired in patients with malignant mesothelioma, which may be related to the pathology of this disease. Our study results and discussion are summarized in Figure 6.

## 11. Conclusion

Our overall findings highlight the following points. (1) Exposure to asbestos has the potential to suppress the function of NK cells and CTLs. (2) It is possible that analysis of immunological parameters, such as NKp46 expression, might contribute to the evaluation of health conditions in asbestos-exposed individuals. (3) CD8^+^ lymphocytes in individuals with pleural plaque may be stimulated by some kind of “non-self” cells. (4) CTL function is impaired in patients with malignant mesothelioma in comparison to plaque-positive individuals. Following these studies, we have continued to examine the effect of asbestos exposure on immune function and analyze specimens from asbestos-exposed individuals and mesothelioma patients. We hope that our studies will contribute to a greater understanding of asbestos exposure-related health disorders, including malignant mesothelioma, in order to improve the cure rate of those diseases.

## Figures and Tables

**Table 1:** The major part of our previous studies about immunological effects of asbestos exposure and analysis for immune functions of patients.

Analyses for	Asbestos in cultures or name of diseases	Results	References
(i) Natural killer (NK) cells			
Human NK cell line, YT-A1	Culture with chrysotile	Decreases in natural cytotoxicity, cell surface NKG2D, and 2B4 and phosphorylation of ERK	[5, 6]
Peripheral blood CD56^+^ NK cells	Malignant mesothelioma	Low cytotoxicity, low expression of cell surface NKp46	[5]
Human NK cells in PBMC	Culture with chrysotile	Decrease in cell surface NKp46	[5]

(ii) T helper cells			
Human T cell line, MT-2	Culture with chrysotile	Resistance against asbestos-induced apoptosis, increases in secretion of IL-10 and expression of bcl-2 mRNA, decreases in secretion of IFN-*γ*, TNF-*α*, IL-6, and CXCL10, and surface expression and mRNA of CXCR3	[1, 2]
	Culture with crocidolite	Resistance against asbestos-induced apoptosis, increases in secretion of IL-10 and ratio of bcl-2/bax mRNAs, and decreases in secretion of IFN-*γ* and TNF-*α*	[4]
Peripheral blood CD4^+^ T cells	Malignant mesothelioma	Very low expression of cell surface CXCR3, low IFN-*γ* mRNA, and high bcl-2 mRNA	[1, 3]
	Pleural plaque	Low expression of cell surface CXCR3	[3]
Isolated human CD4^+^ T cells	Culture with chrysotile	Decreases in cell surface CXCR3 and intracellular IFN-*γ*	[3]

(iii) Cytotoxic T lymphocytes (CTL)			
Human CD8^+^ T cells in mixed lymphocyte reaction	Culture with chrysotile	Decreases in allogeneic cytotoxicity and intracellular IFN-*γ* and granzyme B	[7]
Peripheral blood CD8^+^ T cells	Malignant mesothelioma	High percentage of perforin^+^ cells, stimulation-induced decrease in perforin^+^ cells	[9]
	Pleural plaque	High percentage of perforin^+^ cells	[9]
